# FT011, a Novel Cardiorenal Protective Drug, Reduces Inflammation, Gliosis and Vascular Injury in Rats with Diabetic Retinopathy

**DOI:** 10.1371/journal.pone.0134392

**Published:** 2015-07-29

**Authors:** Devy Deliyanti, Yuan Zhang, Fay Khong, David R. Berka, David I. Stapleton, Darren J. Kelly, Jennifer L. Wilkinson-Berka

**Affiliations:** 1 Department of Immunology and Pathology, Monash University, Melbourne, Victoria, Australia, 3004; 2 Department of Medicine, St Vincent’s Hospital, The University of Melbourne, Fitzroy, Victoria, Australia, 3065; 3 The Florey Institute of Neuroscience and Mental Health, The University of Melbourne, Parkville, Victoria, Australia, 3052; Indiana University College of Medicine, UNITED STATES

## Abstract

Diabetic retinopathy features inflammation as well as injury to glial cells and the microvasculature, which are influenced by hypertension and overactivity of the renin-angiotensin system. FT011 is an anti-inflammatory and anti-fibrotic agent that has been reported to attenuate organ damage in diabetic rats with cardiomyopathy and nephropathy. However, the potential therapeutic utility of FT011 for diabetic retinopathy has not been evaluated. We hypothesized that FT011 would attenuate retinopathy in diabetic Ren-2 rats, which exhibit hypertension due to an overactive extra-renal renin-angiotensin system. Diabetic rats were studied for 8 and 32 weeks and received intravitreal injections of FT011 (50 μM) or vehicle (0.9% NaCl). Comparisons were to age-matched controls. In the 8-week study, retinal inflammation was examined by quantitating vascular leukocyte adherence, microglial/macrophage density and the expression of inflammatory mediators. Macroglial Müller cells, which exhibit a pro-inflammatory and pro-angiogenic phenotype in diabetes, were evaluated in the 8-week study as well as in culture following exposure to hyperglycaemia and FT011 (10, 30, 100 μM) for 72 hours. In the 32-week study, severe retinal vasculopathy was examined by quantitating acellular capillaries and extracellular matrix proteins. In diabetic rats, FT011 reduced retinal leukostasis, microglial density and mRNA levels of intercellular adhesion molecule-1 (ICAM-1). In Müller cells, FT011 reduced diabetes-induced gliosis and vascular endothelial growth factor (VEGF) immunolabeling and the hyperglycaemic-induced increase in ICAM-1, monocyte chemoattractant protein-1, CCL20, cytokine-induced neutrophil chemoattractant-1, VEGF and IL-6. Late intervention with FT011 reduced acellular capillaries and the elevated mRNA levels of collagen IV and fibronectin in diabetic rats. In conclusion, the protective effects of FT011 in cardiorenal disease extend to key elements of diabetic retinopathy and highlight its potential as a treatment approach.

## Introduction

The prognosis for young people diagnosed with diabetic retinopathy (DR) remains poor, and this is related primarily to the progressive damage that occurs in the retina over decades of diabetes mellitus [1]. DR has a considerable global prevalence with approximately 93 million affected people, a number which is expected to escalate in coming years [1]. Current treatments such as laser photocoagulation and anti-angiogenic agents are used to reduce the severe microvascular and tissue damage that occurs in the end-stages and proliferative form of DR. Although these treatments are somewhat successful, they do not prevent the advancement of DR from its early to late stages. It is clear that preventative treatments are urgently required.

DR involves alterations to the microvasculature as well as neurons and glia [2]. The close anatomical arrangement of these cell types means that damage to one cell population influences the health of another [2]. An important early pathological event is inflammation, which presents as increased adherence of leukocytes to the retinal vasculature [3, 4], as well as activation of microglia, the resident immunocompetent cell of the retina, resulting in the release of damaging cytokines [5, 6]. Macroglial Müller cells have an integral role in retinal homeostasis and become gliotic and pro-inflammatory early in DR which accompanied by the increased production of vascular endothelial growth factor (VEGF), compromises the integrity of the blood-retinal barrier [7, 8]. Apoptosis of capillary pericytes is an early feature of DR and followed later by the death of endothelial cells to result in acellular capillaries and under perfused tissue that is the stimulus for clinically observable neovascularization and vascular leakage that characterizes proliferative DR.

The mechanisms involved in the pathogenesis of DR are not completely understood and include oxidative stress, advanced glycation end-products and activation of protein kinase C [9–11]. Overactivity of the renin-angiotensin system (RAS) resulting in elevated levels of angiotensin II and aldosterone, play a key role in stimulating many of the aforementioned pathways in DR [12, 13]. The circulating RAS is associated with the development of DR [14], although the existence of a retinal RAS, which is modulated in diabetes, is likely to be involved [12, 15, 16].

Tranilast is an anti-fibrotic agent used for the treatment of hypertrophic scars and keloid skin disorders, whose mechanisms of action include the reduction of TGF-β and platelet derived growth factor [17]. Previous studies by our group have shown that tranilast reduces fibrosis in the heart and kidney of diabetic rats [18, 19]. Emerging evidence extends the benefits of tranilast to the attenuation of inflammation and vascular injury in organs including ocular tissues [20, 21]. We previously reported the synthesis of a series of cinnamoyl anthranilate derivatives of tranilast, which led to the generation of several derivatives with superior potency and reduced cellular toxicity relative to tranilast [22]. We showed that one of the derivatives, 3- methoxy-4- propargyloxycinnamoyl anthranilate (FT011M, Fibrotech Therapeutics, Melbourne, Australia) improves renal and cardiac function and structure in diabetic animals. The protective effects of FT011M were related to the attenuation of TGF-β and extracellular signal regulated kinase 1/2 activity, the deposition of extracellular matrix proteins as well as inflammation including the infiltration of macrophages [23, 24]. These findings led us to hypothesize that FT011M would provide protection against the retinal inflammation and gliosis that occurs in the early stages of DR as well as the vascular injury that develops with long-standing diabetes. Our hypothesis was examined in the transgenic (mRen)27 (Ren-2) rat which exhibits overactivity of the RAS and rapid onset DR [13, 15, 25–27] as well as primary cultures of retinal Müller cells. Our studies revealed that FT011 has potent protective effects against the development of DR which involves the modulation of inflammatory and angiogenic pathways.

## Materials and Methods

### Animals

This study was carried out in strict accordance with the recommendations in the Guide for the Care and Use of Laboratory Animals of the National Institutes of Health. Procedures adhered to guidelines of the National Health and Medical Research Council (NHMRC) of Australia’s Code for the Care and Use of Animals for Scientific Purposes. The protocol was approved by the Committee on the Ethics of Animal Experiments of The University of Melbourne, St Vincent’s Hospital, Fitzroy, Australia (Permit Number: 055/10). All surgery was performed under sodium pentobarbital anaesthesia, and all efforts were made to minimize suffering. All rats received normal rat chow (Certified Rodent Diet #5002, LabDiet, USA) and drinking water *ad libitum*, and were housed at 22 ± 1°C with a 12-h light/dark cycle.

Following an overnight fast, 6 week old female heterozygous Ren-2 rats (150 to 200g) assigned to become diabetic received by tail vein injection, 55 mg/kg of streptozotocin (STZ, Sigma-Aldrich, St. Louis, USA) diluted in 0.1M citrate buffer, pH 4.5. In order to evaluate the pathological events that occur in the early (inflammation and gliosis) and late (acellular capillaries) stages of DR, rats were randomized to 8 week or 32 week studies. To determine the direct effects of FT011 on retinal cellular pathology, diabetic rats received into each eye intravitreal injections (1 μl) of vehicle or FT011 meglamine salt (FT011M, 50 μM). Non-diabetic rats received into each eye intravitreal injections (1 μl) of vehicle (0.9% NaCl). Intravitreal injections occurred at 2 days and 4 weeks in the 8-week study groups, and at 16, 20, 24 and 28 weeks in the 32-week study groups. The intravitreal injections were performed according to a published method [28]. Rats were anaesthetised with isoflurane inhalation and using a Hamilton syringe, a 31-gauge needle was inserted 1 to 2 mm into the eye, 2 mm behind the limbus at a 45° downward angle to avoid injecting into the lens. The dose of FT011M was based on previous studies in cell culture using multiple cell lines where FT011M administered at a dose range of 10 to 100 μM inhibited cell proliferation and collagen synthesis [23, 24]. Each week, rats were weighed and their blood glucose levels measured (Accu-check Advantage II Blood Glucose Monitor, Roche Diagnostics, USA). Only rats with blood glucose levels >15 mmol/L were considered diabetic. Insulin was administered three times per week to reduce mortality and promote weight gain (2 to 4 units i.p., Humulin NPH, Eli Lilly and Co., Indianapolis, IN, USA). At the end of the studies, rats received an anaesthetic overdose of pentobarbitone sodium (Lethabarb, 60 mg/kg, Virbac, NSW, Australia) and all efforts were made to minimize suffering.

### Vascular leukocyte adhesion

Using an established method, rats were perfused via the right atrium with 0.1M PBS, pH 7.4 to remove non-adherent blood cells, followed by rhodamine-conjugated Concanavalin A lectin (0.5 mg/kg, cat# RL1002, Vector Laboratories, Inc., CA, USA) to stain the vasculature and adherent leukocytes [13, 29]. Eyes were fixed in 4% paraformaldehyde in PBS for 30 min and retina flat-mounted on microscope slides. The vasculature was observed using an Olympus BX51 fluorescent microscope (Olympus, Tokyo, Japan). Non-overlapping images were captured and the number of leukocytes per retina counted at 200x magnification. Five to six rats per group were evaluated.

### Immunohistochemistry for microglia

Ionized calcium binding adaptor protein 1 (Iba1) was used to identify microglia as previously described [29, 30]. Three-μm paraffin sections were incubated overnight at 4°C with Iba1 (1:1000, cat# 019–19741,Wako, Tokyo, Japan). A negative control without the primary antibody and an isotype IgG control was included. Sections were washed with PBS, incubated for 30 min with biotin-conjugated goat anti rabbit IgG (1:200, cat# E0432, DakoCytomation, Glostrup, Denmark), washed again and then incubated with the Vectastain ABC standard kit (Vector Laboratories) for 30 to 45 min and liquid DAB+substrate chromagen system (Dakocytomation) for 15 sec. The sections were counterstained with Harris’ Haematoxylin for 7 min and coverslipped. For quantitation, 4 sections at least 60 μm apart were randomly selected from each eye. In each section, 4 non-overlapping fields of equal dimensions and spanning the entire retina were captured at x400 magnification using a Spot digital camera (SciTech, VIC, Australia). Image J software was used to set a threshold for immunolabeling which was applied to all fields. Immunolabeling was quantitated in the inner retina, which comprised layers from the retinal surface to the inner nuclear layer. Data are presented as the percentage of Iba1 immunolabelling per field of inner retina. Four to 6 rats per group were evaluated.

### Müller cell gliosis

Injury to Müller cells was evaluated with immunohistochemistry for glial fibrillary acidic protein (GFAP) using an established method [30]. Briefly, 3 μm paraffin sections were incubated overnight at 4°C with a rabbit polyclonal anti-GFAP antibody (1:500, cat# Z0334, DakoCytomation). The sections were then washed with PBS and incubated for 45 min with Alexa Flour 488-conjugated goat anti-rabbit IgG (1:200, cat# A-11008, Life Technologies, VIC, Australia), washed again and coverslipped. For quantitation, 4 sections at least 60μm apart were randomly selected from each eye. In each section, 12 non-overlapping fields spanning the mid, central and peripheral areas of retina were captured at x400 magnification using a Spot digital camera (SciTech, VIC, Australia). Image J software was used to set a threshold for immunolabeling which was applied to all fields. Data are presented as the percentage of GFAP immunolabelling per total retinal area in the field. Quantitation was performed in all layers of the retina as well as the mid, central and peripheral retina [30]. Four to 6 rats per group were evaluated.

### VEGF immunolabeling

Immunohistochemistry for VEGF was performed on paraffin sections as previously described [29, 30]. The method described for Iba1 immunolabeling was followed except that the primary antibody was goat anti-rat VEGF164 (1:50, cat# AF564, R&D systems, Minneapolis, Minnesota, USA) and the secondary antibody, biotinylated donkey anti-goat IgG antibody (1:500, cat# 705-065-003, Jackson Immunoresearch, West Grove, Pennsylvania, USA). For quantitation, 4 sections at least 60μm apart were randomly selected from each eye. In each section, 4 non-overlapping fields of equal dimensions and spanning the entire retina were captured at x400 magnification using a Spot digital camera (SciTech, VIC, Australia) and analysed using the Analytical Imaging software (version 6.0, Ontario, Canada). Data are presented as the percentage of VEGF immunolabelling per total field of retina. Five to 6 rats per group were evaluated.

### Cultured Müller cells

To evaluate in more depth the effects of FT011M on Müller cell injury; primary cultures from neonatal Sprague Dawley rats were prepared as previously published [30]. Müller cells were exposed to normoglycaemia (5.5 mmol/L D-glucose and 20 mmol/L mannitol), hyperglycaemia (25 mM D-glucose) and treated with FT011M (10, 30 and 100 μM) for 72 hours. Supernatants were harvested and a rat protein cytokine array performed according to the manufacturers instructions (Cat# ARY008, R&D Systems). The level of monocyte chemoattractant protein-1 (MCP-1) was measured in supernatant using a commercially available rat MCP-1 ELISA kit (Cat# 555130, BD Biosciences, San Jose, CA, USA).

### Acellular capillaries

According to our previous method [13, 27], eyes were fixed overnight in 2% Carson’s fixative and washed in 0.2 mol/l Tris buffer for 4 days. The retina was digested in 1% trypsin solution (Sigma-Aldrich) at 37°C for 1 h and mounted on silane-coated slides. The slides were stained with 1% Periodic acid-Schiff’s reagent (CN Biomedicals Inc., Aurora, OH, USA) and Mayer’s Hematoxlyin (Amber Scientific, WA, Australia). Images from the entire retina were captured at X40 magnification. Acellular capillaries were counted in the central, mid and peripheral retina. Approximately 80 high-power (X40 objective) non-overlapping photomicrographs were taken from each retina (15 central, 30 mid, 35 peripheral regions). Eight to 11 rats per group were evaluated.

### Quantitative RT-PCR

Retinal mRNA levels were determined by quantitative PCR as previously described [13, 31]. One μg of total RNA extracted from retina was DNAse-treated and reverse transcribed and levels of intercellular adhesion molecule-1 (ICAM-1), collagen IV, and fibronectin. mRNA was normalised to 18S rRNA endogenous control and the relative fold difference in expression calculated using the 2^−ΔΔ^CT method. Primers for ICAM-1 are as previously published [13]. Primers for collagen IV and fibronectin are as follows. Fibronectin forward 5'ACTATGACATCAGCGTTATCACTCTCA3”. Fibronectin reverse 5'TAGTGTCCGGACCGATATTGG3'. ColIagen IV forward 5'CATCCGGCCCTTCATTAGC3'. ColIagen IV reverse 5'GACTGTGCACCGCCATCAC3'. Eight to nine rats per group were studied.

### Statistics

Four or more animals per group for each endpoint were evaluated. Data were first assessed for normality by Kolmogorov-Smirnov, D’Agostinos and Pearson ormnibus, as well as Shapiro-Wilk normality tests. Analysis was then performed by one-way ANOVA followed by Bonferroni pro-test analysis (for data that passed normality tests) or by nonparametric Kruskal-Wallis test followed by Dunns post-test (for data that did not pass normality tests). For comparison between two groups, either student t-test or Mann-Whitney tests were used, on data that passed and failed normality tests, respectively. P < 0.05 was considered significant. Investigators were masked to the experimental groups.

## Results

### Body weight and blood glucose

The findings are summarized in Table 1. Diabetic Ren-2 rats did not gain as much body weight as age-matched non-diabetic Ren-2 rats and treatment with FT011M had no effect on body weight gain. Blood glucose levels were elevated in diabetic Ren-2 rats compared to non-diabetic controls and FT011M had no effect on blood glucose levels.

**Table 1:** Animal characteristics.

Group	Body Weight (g)		Blood Glucose (mmol/L)	
**Weeks**	**8**	**32**	**8**	**32**
Non-diabetic control+vehicle	283 ± 5	353 ± 12	8 ± 0.5	8 ± 0.2
Diabetic+vehicle	253 ± 4*	305 ± 5*	32 ± 0.3	31 ± 0.8*
Diabetic+FT011M	261 ± 4*	302 ± 5*	32 ± 0.5	30 ± 1.3*

*P < 0.05 versus control. N = 8 rats per group. Values are mean ± SEM.

### FT011M reduced retinal leukostasis in Ren-2 rats diabetic for 8 weeks

**Fig 1:**
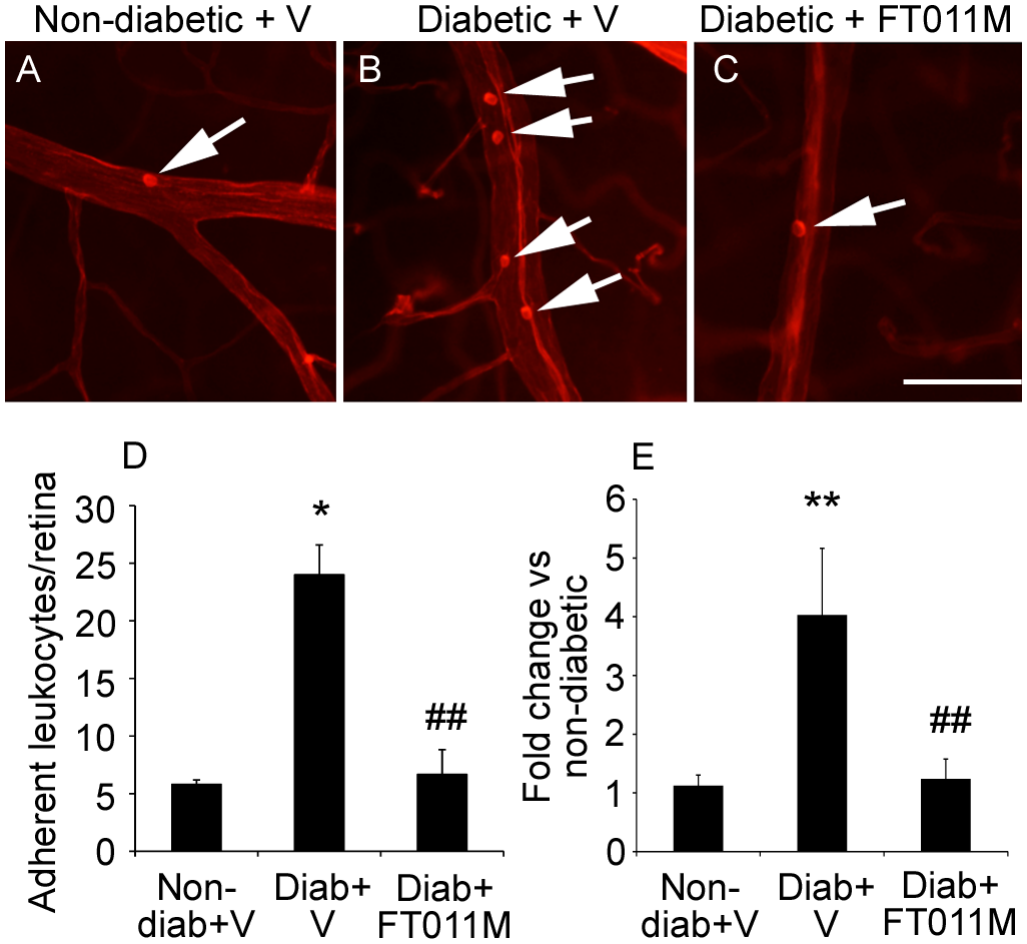
FT011M reduced retinal leukostasis and ICAM-1 mRNA levels in Ren-2 rats diabetic for 8 weeks. Non-diab, non-diabetic. Diab, diabetic. V, vehicle. (A to C) Micrographs showing leukocytes (arrows) adherent to the vasculature. Bar, 40 μm. (D) Leukostasis. N = 5 to 6 rats per group. (E) ICAM-1 mRNA levels. N = 7 to 9 rats per group. *P < 0.05 and **P < 0.01 to non-diab + V. ##P < 0.01 to diab + V. Values are Mean ± SEM.

Retinal leukostasis and the mRNA levels of ICAM-1 were increased in diabetic Ren-2 rats compared to age-matched non-diabetic controls (Fig 1). In diabetic Ren-2 rats, FT011M reduced retinal leukostasis and ICAM-1 to the level of non-diabetic controls (Fig 1).

### FT011M reduced Iba1 immunolabeling of microglia in the retina of Ren-2 rats diabetic for 8 weeks

**Fig 2:**
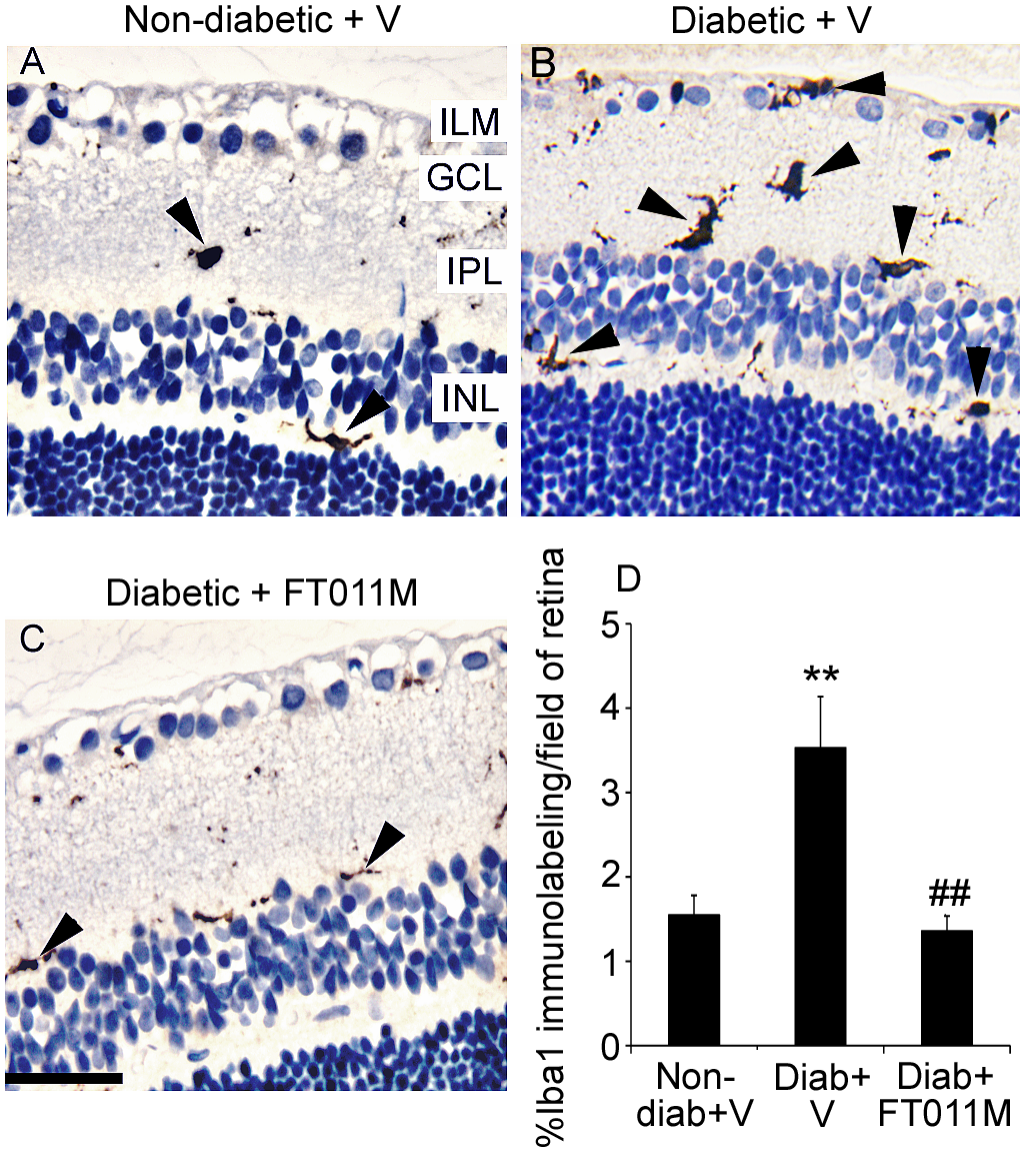
FT011M reduced Iba1-immunolabeled microglia in the retina of Ren-2 rats diabetic for 8 weeks. Non-diab, non-diabetic. Diab, diabetic. V, vehicle. Three-μm paraffin sections. ILM, inner limiting membrane. GCL, ganglion cell layer. IPL, inner plexiform layer. INL, inner nuclear layer. Counterstain, haematoxylin. Original magnification, 400X. Bar, 40 μm. (A to C) In diab + V, Iba1 immunolabeling (arrowheads) was increased compared to non-diab + V. In diabetic rats, FT011M reduced Iba1 immunolabeling to the level of non-diab + V. (D) **P < 0.01 to non-diab + V. ##P < 0.01 to diab + V. N = 4 to 6 rats per group. Values are Mean ± SEM.

In non-diabetic Ren-2 rats, sparse Iba1 immunolabeling was observed in the retina (Fig 2). By contrast, in diabetic Ren-2 rats, extensive Iba1 immunolabeling was detected and associated with blood vessels. FT011M treatment to diabetic rats reduced Iba1 immunolabeling to the level of non-diabetic controls (Fig 2).

### FT011M reduced Müller cell gliosis in Ren-2 rats diabetic for 8 weeks

**Fig 3:**
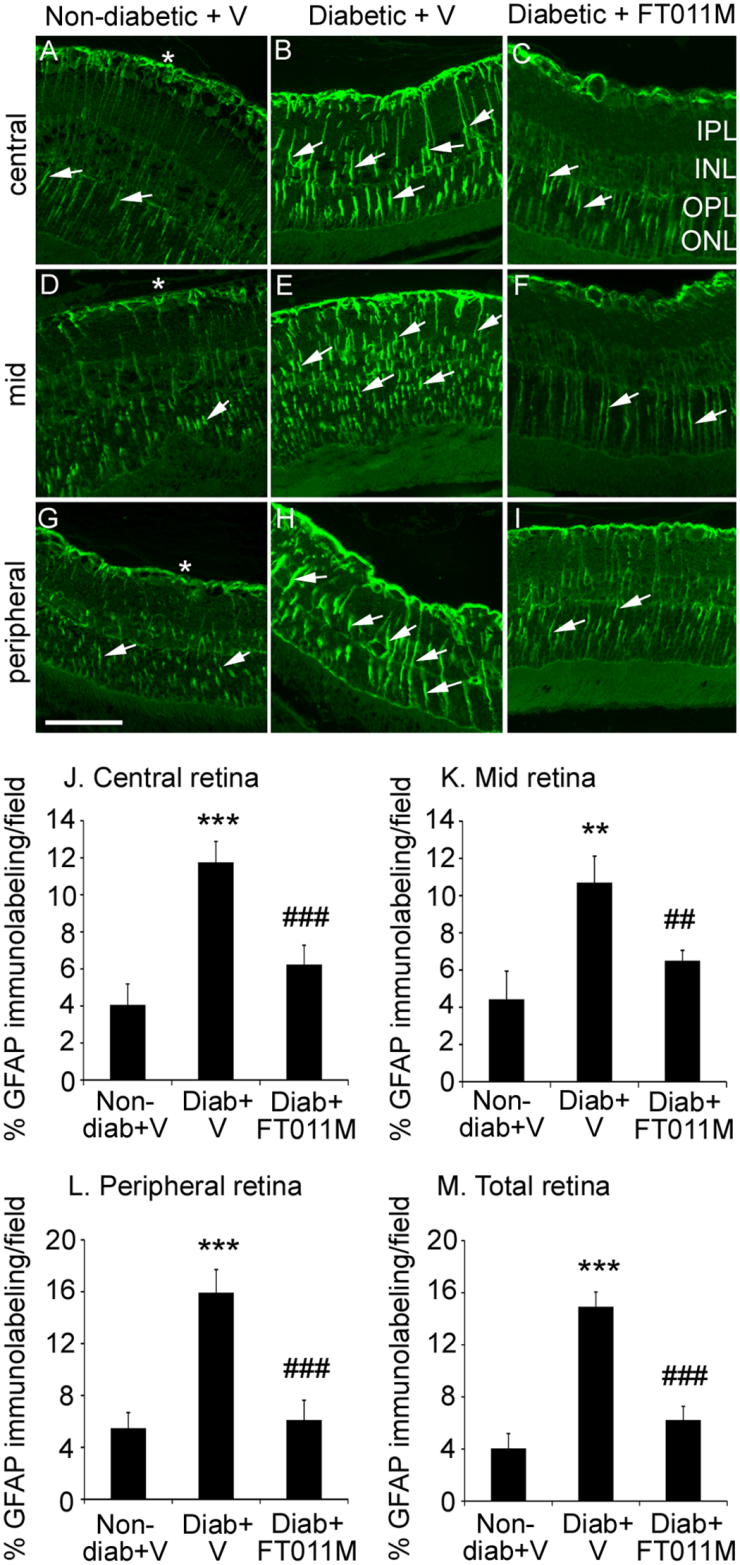
FT011M reduced Müller cell gliosis in the retina of Ren-2 rats diabetic for 8 weeks. Non-diab, non-diabetic. Diab, diabetic. V, vehicle. Three-μm paraffin sections. IPL, inner plexiform layer. INL, inner nuclear layer. OPL, outer plexiform layer. ONL, outer nuclear layer. Original magnification, 400X. Bar, 40 μm. (A to C) Central retina. (D to F) Mid retina. (G to I) Peripheral retina. In non-diab + V, GFAP immunolabeling is present on the retinal surface (asterisk) and in Müller cell processes (arrows) extending throughout the retinal layers and in the central, mid and peripheral retina (A, D, G). GFAP immunolabeling is increased in diab + V (B, E, H). In diabetic rats, FT011M reduced GFAP immunolabeling in the central (G), mid (F) and peripheral (I) retina to the level of non-diab + V. (J to M) *P < 0.01 and ***P < 0.001 to non-diab + V. ##P < 0.01 and ###P < 0.001 to diab + V. N = 4 to 6 rats per group. Values are Mean ± SEM.

In non-diabetic rats, GFAP immunolabeling was present at the retinal surface and in Müller cells processes (Fig 3). In untreated diabetic Ren-2 rats, GFAP immunolabeling in Müller cells was markedly increased in the central, mid and peripheral retina compared to age-matched non-diabetic controls (Fig 3). In diabetic Ren-2 rats, FT011M reduced GFAP immunolabeling in all layers and regions of the retina compared to untreated diabetic Ren-2 rats (Fig 3).

### FT011M reduced VEGF expression in in Ren-2 rats diabetic for 8 weeks

**Fig 4:**
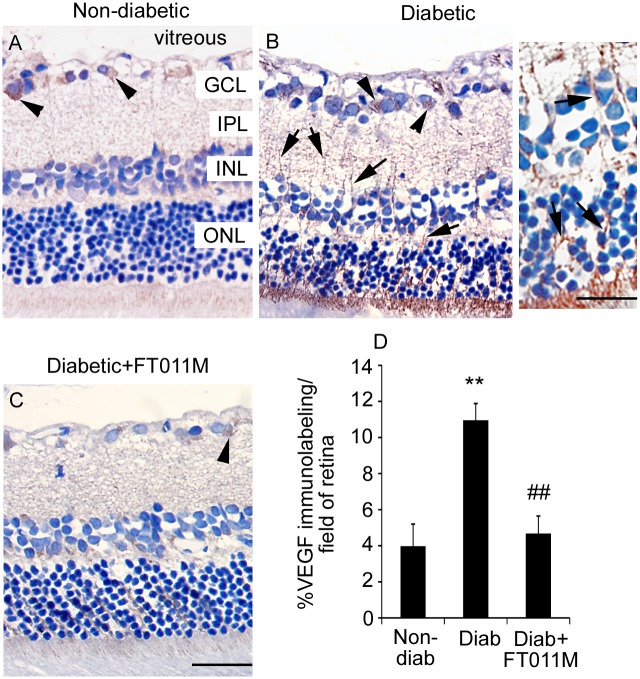
FT011M reduced VEGF immunolabeling in retina of Ren-2 rats diabetic for 8 weeks. Non-diab, non-diabetic. Diab, diabetic. V, vehicle. Three-μm paraffin sections. GCL, ganglion cell layer. IPL, inner plexiform layer. INL, inner nuclear layer. ONL, outer nuclear layer. Counterstain, haematoxylin. (A). In non-diab + V, VEGF immunolabeling is occasionally detected in ganglion cells (arrowheads) in the GCL. (B) In diab + V, VEGF immunolabeling is increased compared to non-diab + V and detected in ganglion cells and Müller cell processes (arrows) extending throughout the retina. Inset showing higher powered image of Müller cell processes. (C) In diabetic rats treated with FT011M, VEGF immunolabeling was reduced to the level of non-diab + V. (A to C) Original magnification, 400X. Bar, 40 μm. Inset: Bar, 75 μm. (D) **P < 0.01 to non-diab + V. ##P < 0.01 to diab + V. N = 4 to 6 rats per group. Values are Mean ± SEM.

In non-diabetic Ren-2 rats immunolabeling for VEGF was detected in occasional ganglion cells, whilst in untreated diabetic Ren-2 rats, VEGF immunolabeling was increased and present in ganglion cells and Müller cell processes extending throughout the retina (Fig 4). The treatment of diabetic Ren-2 rats with FT011M reduced VEGF immunolabeling in ganglion cells and Müller cells (Fig 4).

### FT011M reduced inflammatory and angiogenic mediators in cultured Müller cells

**Fig 5:**
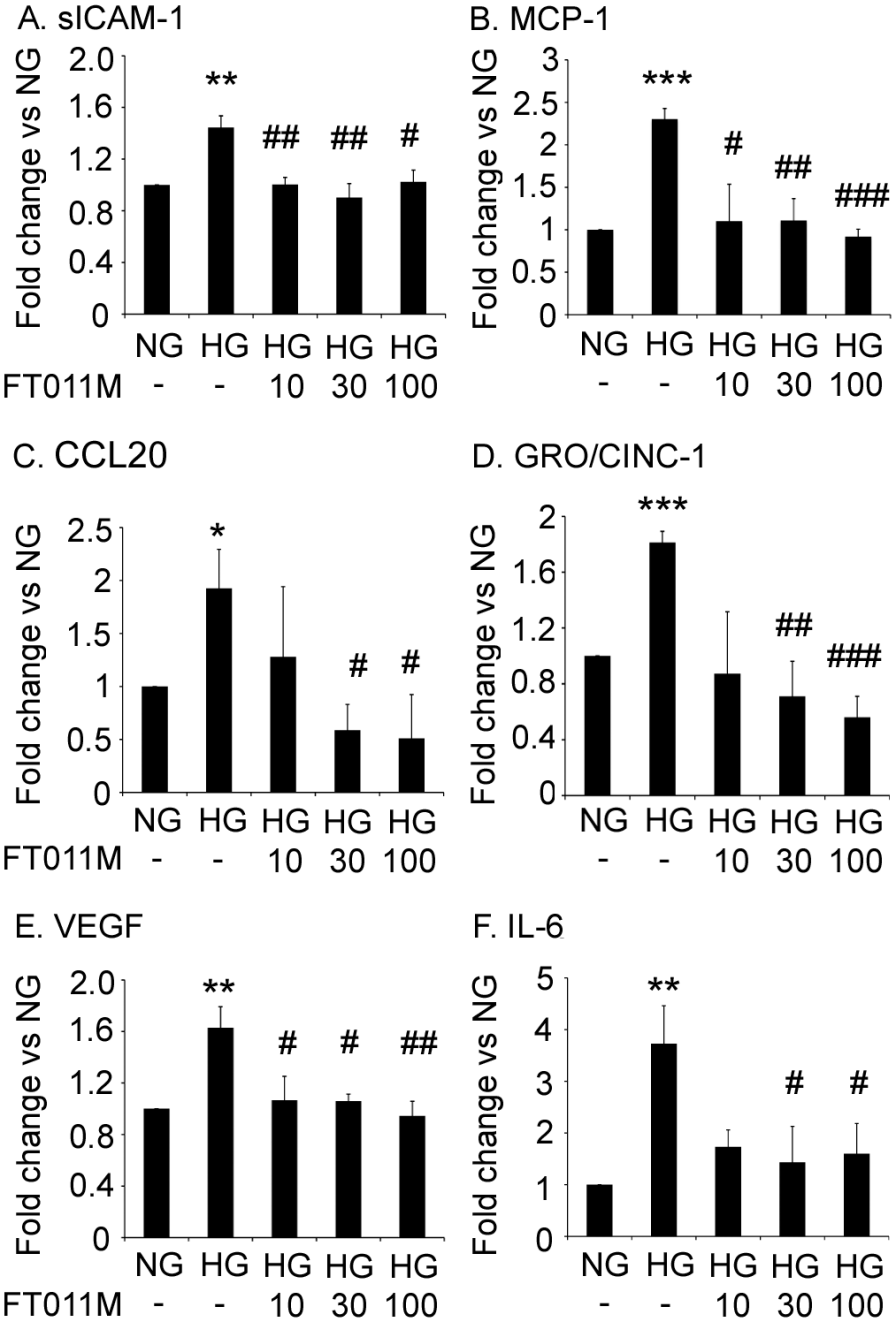
FT011M reduced the protein levels of pro-inflammatory and pro-angiogenic factors in Müller cells. NG, normoglycaemia. HG, hyperglycaemia. (A) soluble ICAM-1 (sICAM-1). (B) MCP-1. (C) CCL20. (D) GRO/CINC-1. (E) VEGF. (F) IL-6. (A, C, D, E and F) values are arbitrary units for cytokine array. Values for (B) are pg/ml for ELISA. *P < 0.05, **P < 0.01 and ***P < 0.001 to untreated (-) NG. #P < 0.05, ##P < 0.01 and ###P < 0.001 to untreated (-) HG. Experiments performed in triplicate with 3 samples per experiment. Values are Mean ± SEM.

In Müller cells exposed to hyperglycaemia, the protein levels of MCP-1, soluble ICAM-1, CCL20, growth-regulated oncogene/cytokine-induced neutrophil chemoattractant-1 (GRO/CINC-1), VEGF and IL-6 were increased compared to normoglycaemic controls (Fig 5). FT011M at concentrations of 10, 30 and 100 μM, reduced the hyperglycaemic-induced increased in these factors, except for CCL20 and IL-6, which were reduced with 30 and 100 μM FT011M (Fig 5).

### FT011M reduced acellular capillaries and the expression of extracellular matrix proteins in Ren-2 rats diabetic for 32 weeks

**Fig 6:**
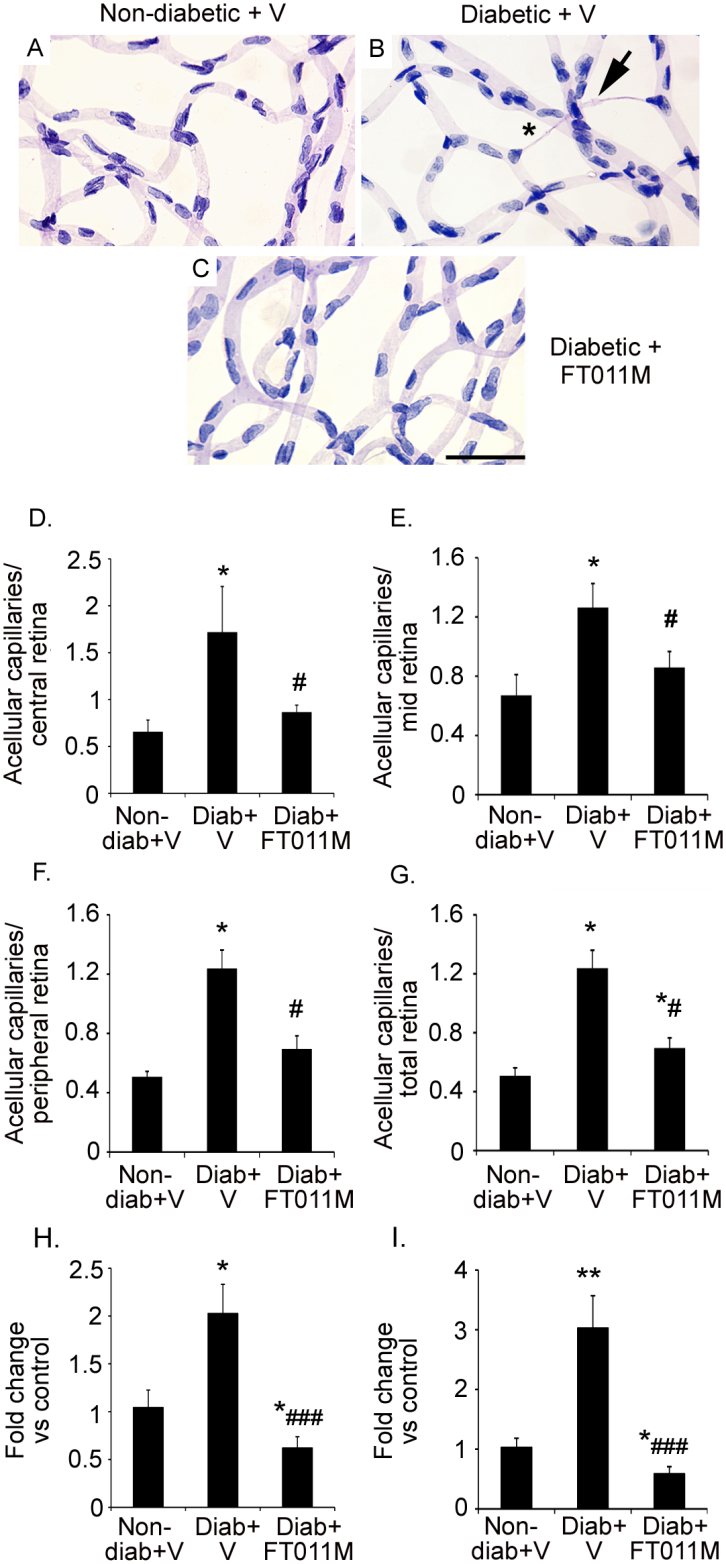
FT011M reduced acellular capillaries and extracellular matrix proteins in retina from Ren-2 rats diabetic for 32 weeks. Non-diab, non-diabetic. Diab, diabetic. V, vehicle. Stain, Periodic acid-Schiff’s reagent. Bar, 40 μm. (A to C) In diab + V, acellular capillaries (asterisk) were increased in all regions of retina compared to non-diabetic + V. In diabetic rats, FT011M reduced acellular capillaries in all regions of the retina. Arrow, pericyte ghost. (D to G) *P < 0.05 to non-diab + V. #P < 0.05 to diab + V. N = 8 to 11 rats per group. (H) Collagen IV mRNA levels in retina. (I) Fibronectin mRNA levels in retina. *P < 0.05, **P < 0.01 to non-diab + V. ###P < 0.001 to diab + V. N = 5 to 9 rats per group. Values are Mean±SEM.

In untreated diabetic Ren-2 rats the number of acellular capillaries in the retina was increased compared to age-matched non-diabetic controls (Fig 6). In diabetic Ren-2 rats treated with FT011M, acellular capillaries were reduced in the central, mid, peripheral and total retina compared to untreated diabetic Ren-2 rats (Fig 6). In the retina of diabetic Ren-2 rats the mRNA levels of the extracellular matrix proteins, collagen IV and fibronectin were increased compared to non-diabetic controls (Fig 6). In diabetic Ren-2 rats treated with FT011M, collagen IV and fibronectin mRNA levels in retina were reduced to below the levels of non-diabetic controls (Fig 6).

## Discussion

The present study demonstrates that FT011M attenuates key features of DR including retinal inflammation and Müller cell gliosis and secretion of inflammatory and vascular mediators as well as acellular capillaries. The retino-protective effects of FT011M were tested in the diabetic Ren-2 rat, as this animal is a robust model of rapid onset and severe diabetic complications including cardiomyopathy, nephropathy and retinopathy [15, 32, 33]. This organ pathology is not driven by the hypertension that develops in the Ren-2 rat, but rather an amplified RAS in tissues, excluding the kidney, due to expression of the mouse Ren-2 gene [25]. Indeed, RAS components such as renin and prorenin are increased in the retinas of Ren-2 rats compared to their genetic control, the Sprague Dawley rat [15, 34]. This notion was confirmed in our previous studies where both angiotensin II and β-adrenergic receptor inhibition reduced hypertension in the Ren-2 rat, but only angiotensin II blockade improved DR [26, 27]. The pathogenic effects of the RAS in retina is established and involves the stimulation of vascular and neuro-glial pathology and the up-regulation of oxidative stress, advanced glycation end-products and inflammatory pathways [4, 11, 31, 35]. Previous studies in models of nephropathy and cardiomyopathy demonstrated that the oral administration of FT011M reduced organ pathology [23, 24]. To avoid any possible systemic effects of FT011M being the primary influence on DR we used an intravitreal approach for drug delivery. The ability of intraocular delivered FT011M to reduce DR in the setting of an amplified tissue RAS and advanced retinal pathology, highlights the potential of FT011M as a treatment approach for DR.

DR has features of chronic sub-clinical inflammation that contribute to vascular and neuro-glial cell pathology [4, 36]. Studies in diabetic patients and monkeys revealed increased numbers of neutrophils in retinal and choroidal blood vessels as well as amplified expression of leukocyte adhesion molecules such as ICAM-1 [3, 37]. Indeed, blockade of ICAM-1 attenuates retinal leukostasis in animal models of DR [38]. The consequences of increased leukostasis in DR are multiple and include the closure of some capillaries as well as the release of injurious cytokines [3, 37]. The ability of FT011M to reduce retinal leukostasis in the diabetic Ren-2 rat as well as the mRNA levels of ICAM-1, suggests FT011M has anti-inflammatory actions that are relevant to DR. The effectiveness of FT011M would appear to be similar to the angiotensin type 1 receptor blocker, candesartan, with both agents normalizing retinal leukostasis in diabetic Ren-2 rats [13]. However, direct comparisons between the two agents should be considered in the context that diabetes-induced retinal leukostasis and DR was more severe in this previous study due to rats being homozygous rather than heterozygous for the Ren-2 gene [13].

Retinal inflammation in DR also involves activation of resident microglia [5, 6]. Under normal circumstances, microglia exert a housekeeping role by constantly patroling the retina and producing anti-inflammatory cytokines and neuroprotective factors in response to cellular damage [39]. Paradoxically, microglia undergo phenotypic changes in DR to become activated and harm retinal cells [6, 39]. The reduction in the diabetes-induced increase in Iba1 immunolabeled microglial following FT011M treatment is consistent with our previous study in which FT011M decreased macrophage infiltration in kidneys from diabetic animals [24]. Together, our findings indicate that FT011M has the capacity to dampen retinal inflammation by reducing leukocyte adhesion to the retinal vasculature and Iba1 immunolabeled microglia in DR. Although the mechanisms by which this occur are not fully understood, the anti-inflammatory properties of FT011M are consistent with those of its parent compound, tranilast, which reduced vascular cellular adhesion molecule-1 expression and the release of certain chemokines in human corneal fibroblasts [21].

Müller cells perform crucial functions in the retina including maintenance of the blood-retinal barrier, neuroprotection and suppression of inflammation [7]. The health of Müller cells can be robustly demonstrated by measuring GFAP, a marker of intermediate filaments [7]. In normoglycemia, GFAP expression is restricted to astrocytes at the retinal surface, whilst in hyperglycaemia and other situations of retinal stress, Müller cells exhibit GFAP along the length of their cell processes [6–8]. Müller cell gliosis occurs within the first few months of diabetes in rats, and precedes the onset of severe microvascular changes [6, 40]. In the present study, non-diabetic Ren-2 rats exhibited gliosis of Müller cells, which is most likely due to the elevated levels of RAS components in the retinas of these animals. Although few studies have directly evaluated the effects of RAS components on Müller cell function in DR, there is evidence that angiotensin II via the angiotensin type 1 receptor promotes Müller cell gliosis [41]. We found that Müller cell gliosis was marked in all regions of the retina (central, mid, peripheral) of Ren-2 rats in early DR compared to non-diabetic controls, which is consistent with the rapid onset and severity of DR that develops in this animal model [15]. A recent study in non-diabetic and diabetic Ren-2 rats, appears to show less severe Müller cell gliosis than demonstrated here, although the GFAP immunolabeling was not quantitated [42]. The differences between the findings of the two studies are not completely clear. However, in the previous study STZ diabetes was induced in Ren-2 rats that were considerably older (400 to 500g) than in our study (150 to 200g), a factor that may have altered the reactivity of Müller cells. Nonetheless, FT011M’s reduction of GFAP immunolabeling in diabetic Ren-2 rats indicates the capacity of this compound to improve the health of a retinal cell type that has a vital role in retinal homeostasis [7].

Müller cells when injured in diabetes exhibit a pro-inflammatory phenotype [7] reflected by increased expression of MCP-1 [43], which acts to attract microglia to the retina [44], and leukocyte adhesion molecules such as ICAM-1 [45]. By using primary cultures we demonstrated FT011M directly dampens the hyperglycaemic-induced inflammatory response in Müller cells by reducing MCP-1 and ICAM-1 protein. Furthermore, FT011M reduced CCL20 and GRO/CINC-1, chemoattractants increased in eyes from diabetic patients [46, 47] and diabetic mice [48]. Diabetes also compromises the ability of Müller cells to maintain the integrity of the blood-retinal barrier and prevent neovascularization by increasing their secretion of the potent permeability and angiogenic factor, VEGF [49, 50]. The ability of FT011M to reduce VEGF in Müller cells was demonstrated in retina from diabetic Ren-2 rats and confirmed in vitro, where the hyperglycaemic-related increase in VEGF protein as well as IL-6, a cytokine which is elevated in vitreous of individuals with DR [51] and induces VEGF transcription in glia [52], was reduced by FT011M. Collectively, these findings indicate a potential role for this compound in attenuating vasculopathy in DR. Severe alterations to the microvasculature occur after long-standing diabetes and include the development of acellular capillaries which precede neovascularization as well as basement membrane thickening [53, 54]. Depending on the rodent model studied, acellular capillaries occur after 6 to 12 months of diabetes [53]. Here, we used a late intervention treatment approach that would be applicable to individuals with established DR. The ability of FT011M to reduce acellular capillaries and the expression of extracellular matrix proteins, collagen IV and fibronectin, in diabetic Ren-2 rats indicates that this compound has vasculo-protective actions that predominate despite the detrimental effects of inflammation and Müller cell gliosis in early DR. FT011M’s effects on the vasculature have not been previously studied, and are consistent with tranilast’s restraint of proliferation, chemotaxis and tubulogenesis of bovine retinal endothelial cells [41] and angiogenesis in the cornea [20]. A limitation of our study is that we were unable to determine if FT011M has anti-angiogenic properties in DR due to the absence of this pathology in diabetic rodents [53]. Further studies in pre-clinical models of retinal angiogenesis are required to elucidate FT011M’s effects on this aspect of vascular injury.

In conclusion, we demonstrated the protective effects of FT011M in diabetic cardiorenal disease and fibrosis extend to DR, where this compound attenuated key features of retinal pathology. Together, our findings indicate the potential of FT011M as an early and late intervention approach for the treatment of DR.

## References

[1] Yau JW, Rogers SL, Kawasaki R, Lamoureux EL, Kowalski JW, Bek T, et al Global prevalence and major risk factors of diabetic retinopathy. Diabetes Care. 2012; 35: 556–64. 10.2337/dc11-1909 22301125 PMC3322721

[2] Antonetti DA, Barber AJ, Bronson SK, Freeman WM, Gardner TW, Jefferson LS, et al Diabetic retinopathy: seeing beyond glucose-induced microvascular disease. Diabetes. 2006; 55: 2401–11. 16936187 10.2337/db05-1635

[3] McLeod DS, Lefer DJ, Merges C, Lutty GA. Enhanced expression of intracellular adhesion molecule-1 and P-selectin in the diabetic human retina and choroid. Am J Pathol. 1995; 147: 642–53. 7545873 PMC1870979

[4] Joussen AM, Poulaki V, Le ML, Koizumi K, Esser C, Janicki H, et al A central role for inflammation in the pathogenesis of diabetic retinopathy. FASEB J. 2004; 18: 1450–2. 15231732 10.1096/fj.03-1476fje

[5] Zeng XX, Ng YK, Ling EA. Neuronal and microglial response in the retina of streptozotocin-induced diabetic rats. Vis Neurosci. 2000; 17: 463–71. 10910112 10.1017/s0952523800173122

[6] Rungger-Brandle E, Dosso AA, Leuenberger PM. Glial reactivity, an early feature of diabetic retinopathy. Invest Ophthalmol Vis Sci. 2000; 41: 1971–80. 10845624

[7] Bringmann A, Pannicke T, Grosche J, Francke M, Wiedemann P, Skatchkov SN, et al Muller cells in the healthy and diseased retina. Prog Retin Eye Res. 2006; 25: 397–424. 16839797 10.1016/j.preteyeres.2006.05.003

[8] Mizutani M, Gerhardinger C, Lorenzi M. Muller cell changes in human diabetic retinopathy. Diabetes. 1998; 47: 445–9. 9519752 10.2337/diabetes.47.3.445

[9] Giacco F, Brownlee M. Oxidative stress and diabetic complications. Circ Res. 2010; 107: 1058–70. 10.1161/CIRCRESAHA.110.223545 21030723 PMC2996922

[10] Curtis TM, Scholfield CN. The role of lipids and protein kinase Cs in the pathogenesis of diabetic retinopathy. Diabetes Metab Res Rev. 2004; 20: 28–43. 14737743 10.1002/dmrr.431

[11] Chen M, Curtis TM, Stitt AW. Advanced glycation end products and diabetic retinopathy. Curr Med Chem. 2013; 20: 3234–40. 23745547 10.2174/09298673113209990025

[12] Wilkinson-Berka JL, Agrotis A, Deliyanti D. The retinal renin-angiotensin system: roles of angiotensin II and aldosterone. Peptides. 2012; 36: 142–50. 10.1016/j.peptides.2012.04.008 22537944

[13] Miller AG, Tan G, Binger KJ, Pickering RJ, Thomas MC, Nagaraj RH, et al Candesartan attenuates diabetic retinal vascular pathology by restoring glyoxalase-I function. Diabetes. 2010; 59: 3208–15. 10.2337/db10-0552 20852029 PMC2992784

[14] Franken AA, Derkx FH, Schalekamp MA, Man in t'Veld AJ, Hop WC, van Rens EH, et al Association of high plasma prorenin with diabetic retinopathy. J Hypertens Suppl. 1988; 6: S461–3. 3071586 10.1097/00004872-198812040-00145

[15] Moravski CJ, Skinner SL, Stubbs AJ, Sarlos S, Kelly DJ, Cooper ME, et al The renin-angiotensin system influences ocular endothelial cell proliferation in diabetes: transgenic and interventional studies. Am J Pathol. 2003; 162: 151–60. 12507898 10.1016/S0002-9440(10)63806-0 PMC1851119

[16] Danser AH, van den Dorpel MA, Deinum J, Derkx FH, Franken AA, Peperkamp E, et al Renin, prorenin, and immunoreactive renin in vitreous fluid from eyes with and without diabetic retinopathy. J Clin Endocrinol Metab. 1989; 68: 160–7. 2642484 10.1210/jcem-68-1-160

[17] Miyazawa K, Kikuchi S, Fukuyama J, Hamano S, Ujiie A. Inhibition of PDGF- and TGF-beta 1-induced collagen synthesis, migration and proliferation by tranilast in vascular smooth muscle cells from spontaneously hypertensive rats. Atherosclerosis. 1995; 118: 213–21. 8770315 10.1016/0021-9150(95)05607-6

[18] Kelly DJ, Zhang Y, Connelly K, Cox AJ, Martin J, Krum H, et al Tranilast attenuates diastolic dysfunction and structural injury in experimental diabetic cardiomyopathy. Am J Physiol Heart Circ Physiol. 2007; 293: H2860–9. 17720766 10.1152/ajpheart.01167.2006

[19] Mifsud S, Kelly DJ, Qi W, Zhang Y, Pollock CA, Wilkinson-Berka JL, et al Intervention with tranilast attenuates renal pathology and albuminuria in advanced experimental diabetic nephropathy. Nephron Physiol. 2003; 95: p83–91. 14694265 10.1159/000074845

[20] Takehana Y, Kurokawa T, Kitamura T, Tsukahara Y, Akahane S, Kitazawa M, et al Suppression of laser-induced choroidal neovascularization by oral tranilast in the rat. Invest Ophthalmol Vis Sci. 1999; 40: 459–66. 9950606

[21] Adachi T, Fukuda K, Kondo Y, Nishida T. Inhibition by tranilast of the cytokine-induced expression of chemokines and the adhesion molecule VCAM-1 in human corneal fibroblasts. Invest Ophthalmol Vis Sci. 2010; 51: 3954–60. 10.1167/iovs.09-4161 20335611

[22] Zammit SC, Cox AJ, Gow RM, Zhang Y, Gilbert RE, Krum H, et al Evaluation and optimization of antifibrotic activity of cinnamoyl anthranilates. Bioorg Med Chem Lett. 2009; 19: 7003–6. 10.1016/j.bmcl.2009.09.120 19879136

[23] Zhang Y, Edgley AJ, Cox AJ, Powell AK, Wang B, Kompa AR, et al FT011, a new anti-fibrotic drug, attenuates fibrosis and chronic heart failure in experimental diabetic cardiomyopathy. Eur J Heart Fail. 2012; 14: 549–62. 10.1093/eurjhf/hfs011 22417655

[24] Gilbert RE, Zhang Y, Williams SJ, Zammit SC, Stapleton DI, Cox AJ, et al A purpose-synthesised anti-fibrotic agent attenuates experimental kidney diseases in the rat. PLoS One. 2012; 7: e47160 10.1371/journal.pone.0047160 23071743 PMC3468513

[25] Mullins JJ, Peters J, Ganten D. Fulminant hypertension in transgenic rats harbouring the mouse Ren-2 gene. Nature. 1990; 344: 541–4. 2181319 10.1038/344541a0

[26] Phipps JA, Wilkinson-Berka JL, Fletcher EL. Retinal dysfunction in diabetic ren-2 rats is ameliorated by treatment with valsartan but not atenolol. Invest Ophthalmol Vis Sci. 2007; 48: 927–34. 17251496 10.1167/iovs.06-0892

[27] Wilkinson-Berka JL, Tan G, Jaworski K, Ninkovic S. Valsartan but not atenolol improves vascular pathology in diabetic Ren-2 rat retina. Am J Hypertens. 2007; 20: 423–30. 17386351 10.1016/j.amjhyper.2006.09.018

[28] Gao H, Pennesi M, Shah K, Qiao X, Hariprasad SM, Mieler WF, et al Safety of intravitreal voriconazole: electroretinographic and histopathologic studies. Trans Am Ophthalmol Soc. 2003; 101: 183–9; discussion 9. 14971576 PMC1358987

[29] Wilkinson-Berka JL, Deliyanti D, Rana I, Miller AG, Agrotis A, Armani R, et al NADPH oxidase, NOX1, mediates vascular injury in ischemic retinopathy. Antioxid Redox Signal. 2014; 20: 2726–40. 10.1089/ars.2013.5357 24053718 PMC4026404

[30] Deliyanti D, Armani R, Casely D, Figgett WA, Agrotis A, Wilkinson-Berka JL. Retinal Vasculopathy Is Reduced by Dietary Salt Restriction: Involvement of Glia, ENaCalpha, and the Renin-Angiotensin-Aldosterone System. Arterioscler Thromb Vasc Biol. 2014; 34: 2033–41. 10.1161/ATVBAHA.114.303792 25012132

[31] Wilkinson-Berka JL, Tan G, Jaworski K, Miller AG. Identification of a retinal aldosterone system and the protective effects of mineralocorticoid receptor antagonism on retinal vascular pathology. Circ Res. 2009; 104: 124–33. 10.1161/CIRCRESAHA.108.176008 19038868

[32] Connelly KA, Kelly DJ, Zhang Y, Prior DL, Martin J, Cox AJ, et al Functional, structural and molecular aspects of diastolic heart failure in the diabetic (mRen-2)27 rat. Cardiovasc Res. 2007; 76: 280–91. 17716638 10.1016/j.cardiores.2007.06.022

[33] Kelly DJ, Wilkinson-Berka JL, Allen TJ, Cooper ME, Skinner SL. A new model of diabetic nephropathy with progressive renal impairment in the transgenic (mRen-2)27 rat (TGR). Kidney Int. 1998; 54: 343–52. 9690200 10.1046/j.1523-1755.1998.00019.x

[34] Moravski CJ, Kelly DJ, Cooper ME, Gilbert RE, Bertram JF, Shahinfar S, et al Retinal neovascularization is prevented by blockade of the renin-angiotensin system. Hypertension. 2000; 36: 1099–104. 11116132 10.1161/01.hyp.36.6.1099

[35] Wilkinson-Berka JL, Rana I, Armani R, Agrotis A. Reactive oxygen species, Nox and angiotensin II in angiogenesis: implications for retinopathy. Clin Sci (Lond). 2013; 124: 597–615. 23379642 10.1042/CS20120212

[36] Zhang W, Liu H, Rojas M, Caldwell RW, Caldwell RB. Anti-inflammatory therapy for diabetic retinopathy. Immunotherapy. 2011; 3: 609–28. 10.2217/imt.11.24 21554091 PMC3671852

[37] Kim SY, Johnson MA, McLeod DS, Alexander T, Hansen BC, Lutty GA. Neutrophils are associated with capillary closure in spontaneously diabetic monkey retinas. Diabetes. 2005; 54: 1534–42. 15855343 10.2337/diabetes.54.5.1534

[38] Miyamoto K, Khosrof S, Bursell SE, Rohan R, Murata T, Clermont AC, et al Prevention of leukostasis and vascular leakage in streptozotocin-induced diabetic retinopathy via intercellular adhesion molecule-1 inhibition. Proc Natl Acad Sci U S A. 1999; 96: 10836–41. 10485912 10.1073/pnas.96.19.10836 PMC17969

[39] McCarthy CA, Widdop RE, Deliyanti D, Wilkinson-Berka JL. Brain And Retinal Microglia In Health And Disease: An Unrecognized Target Of The Renin-Angiotensin System. Clin Exp Pharmacol Physiol. 2013; 40: 571–9. 10.1111/1440-1681.12099 23601050

[40] Barber AJ, Antonetti DA, Gardner TW. Altered expression of retinal occludin and glial fibrillary acidic protein in experimental diabetes. The Penn State Retina Research Group. Invest Ophthalmol Vis Sci. 2000; 41: 3561–8. 11006253

[41] Kurihara T, Ozawa Y, Shinoda K, Nagai N, Inoue M, Oike Y, et al Neuroprotective effects of angiotensin II type 1 receptor (AT1R) blocker, telmisartan, via modulating AT1R and AT2R signaling in retinal inflammation. Invest Ophthalmol Vis Sci. 2006; 47: 5545–52. 17122147 10.1167/iovs.06-0478

[42] Batenburg WW, Verma A, Wang Y, Zhu P, van den Heuvel M, van Veghel R, et al Combined renin inhibition/(pro)renin receptor blockade in diabetic retinopathy—a study in transgenic (mREN2)27 rats. PLoS One. 2014; 9: e100954 10.1371/journal.pone.0100954 24968134 PMC4072720

[43] Zong H, Ward M, Madden A, Yong PH, Limb GA, Curtis TM, et al Hyperglycaemia-induced pro-inflammatory responses by retinal Muller glia are regulated by the receptor for advanced glycation end-products (RAGE). Diabetologia. 2010; 53: 2656–66. 10.1007/s00125-010-1900-z 20835858

[44] Nakazawa T, Hisatomi T, Nakazawa C, Noda K, Maruyama K, She H, et al Monocyte chemoattractant protein 1 mediates retinal detachment-induced photoreceptor apoptosis. Proc Natl Acad Sci U S A. 2007; 104: 2425–30. 17284607 10.1073/pnas.0608167104 PMC1892947

[45] Shelton MD, Distler AM, Kern TS, Mieyal JJ. Glutaredoxin regulates autocrine and paracrine proinflammatory responses in retinal glial (muller) cells. J Biol Chem. 2009; 284: 4760–6. 10.1074/jbc.M805464200 19074435 PMC2643491

[46] Schoenberger SD, Kim SJ, Sheng J, Rezaei KA, Lalezary M, Cherney E. Increased prostaglandin E2 (PGE2) levels in proliferative diabetic retinopathy, and correlation with VEGF and inflammatory cytokines. Invest Ophthalmol Vis Sci. 2012; 53: 5906–11. 10.1167/iovs.12-10410 22871833

[47] Lange CA, Stavrakas P, Luhmann UF, de Silva DJ, Ali RR, Gregor ZJ, et al Intraocular oxygen distribution in advanced proliferative diabetic retinopathy. Am J Ophthalmol. 2011; 152: 406–12.e3. 10.1016/j.ajo.2011.02.014 21723532

[48] He J, Wang H, Liu Y, Li W, Kim D. Blockade of vascular endothelial growth factor receptor 1 prevents inflammation and vascular leakage in diabetic retinopathy. 2015; 2015: 605946. 10.1155/2015/605946 PMC4363713 25821590

[49] Pierce EA, Avery RL, Foley ED, Aiello LP, Smith LE. Vascular endothelial growth factor/vascular permeability factor expression in a mouse model of retinal neovascularization. Proc Natl Acad Sci U S A. 1995; 92: 905–9. 7846076 10.1073/pnas.92.3.905 PMC42729

[50] Wang J, Xu X, Elliott MH, Zhu M, Le YZ. Muller cell-derived VEGF is essential for diabetes-induced retinal inflammation and vascular leakage. Diabetes. 2010; 59: 2297–305. 10.2337/db09-1420 20530741 PMC2927953

[51] Yuuki T, Kanda T, Kimura Y, Kotajima N, Tamura J, Kobayashi I, et al Inflammatory cytokines in vitreous fluid and serum of patients with diabetic vitreoretinopathy. J Diabetes Complications. 2001; 15: 257–9. 11522500 10.1016/s1056-8727(01)00155-6

[52] Loeffler S, Fayard B, Weis J, Weissenberger J. Interleukin-6 induces transcriptional activation of vascular endothelial growth factor (VEGF) in astrocytes in vivo and regulates VEGF promoter activity in glioblastoma cells via direct interaction between STAT3 and Sp1. Int J Cancer. 2005; 115: 202–13. 15688401 10.1002/ijc.20871

[53] Lai AK, Lo AC. Animal models of diabetic retinopathy: summary and comparison. J Diabetes Res. 2013; 2013: 106594 10.1155/2013/106594 24286086 PMC3826427

[54] Stitt A, Gardiner TA, Alderson NL, Canning P, Frizzell N, Duffy N, et al The AGE inhibitor pyridoxamine inhibits development of retinopathy in experimental diabetes. Diabetes. 2002; 51: 2826–32. 12196477 10.2337/diabetes.51.9.2826

